# Neonatal Overfeeding Induced by Small Litter Rearing Causes Altered Glucocorticoid Metabolism in Rats

**DOI:** 10.1371/journal.pone.0025726

**Published:** 2011-11-04

**Authors:** Miao Hou, Yanhua Liu, Lijun Zhu, Bin Sun, Mei Guo, Jonas Burén, Xiaonan Li

**Affiliations:** 1 Department of Children Health Care, Nanjing Medical University, Nanjing Children's Hospital, Nanjing, China; 2 Department of General Surgery, Nanjing Medical University, Nanjing Children's Hospital, Nanjing, China; 3 Institute of Pediatric Research, Nanjing Medical University, Nanjing, China; 4 Department of Public Health and Clinical Medicine, Umeå University, Umeå, Sweden; Karolinska Institutet, Sweden

## Abstract

Elevated glucocorticoid (GC) activity may be involved in the development of the metabolic syndrome. Tissue GC exposure is determined by the tissue-specific GC-activating enzyme 11β-hydroxysteriod dehydrogenase type 1 (11β-HSD1) and the GC-inactivating enzyme 5α-reductase type 1 (5αR1), as well as 5β-reductase (5βR). Our aim was to study the effects of neonatal overfeeding induced by small litter rearing on the expression of GC-regulating enzymes in adipose tissue and/or liver and on obesity-related metabolic disturbances during development. Male Sprague-Dawley rat pup litters were adjusted to litter sizes of three (small litters, SL) or ten (normal litters, NL) on postnatal day 3 and then given standard chow from postnatal week 3 onward (W3). Small litter rearing induced obesity, hyperinsulinemia, and higher circulating corticosterone in adults. 11β-HSD1 expression and enzyme activity in retroperitoneal, but not in epididymal, adipose tissue increased with postnatal time and peaked at W5/W6 in both groups before declining. From W8, 11β-HSD1 expression and enzyme activity levels in retroperitoneal fat persisted at significantly higher levels in SL compared to NL rats. Hepatic 11β-HSD1 enzyme activity in SL rats was elevated from W3 to W16 compared to NL rats. Hepatic 5αR1 and 5βR expression was higher in SL compared to NL rats after weaning until W6, whereupon expression decreased in the SL rats and remained similar to that in NL rats. In conclusion, small litter rearing in rats induced peripheral tissue-specific alterations in 11β-HSD1 expression and activity and 5αR1 and 5βR expression during puberty, which could contribute to elevated tissue-specific GC exposure and aggravate the development of metabolic dysregulation in adults.

## Introduction

The global epidemic of obesity and related metabolic disorders continues to progress at an alarming rate in both adults and children [1], [2]. Clinical and experimental studies have shown an association between the environments during the perinatal period and the development of features of metabolic syndrome, such as hypertension, type 2 diabetes, insulin resistance, and hyperlipidemia later in life [3]. In particular, nutritional status is an important factor. Under- or overnutrition during the prenatal period increases the risk of obesity and hyperinsulinemia [4]. In addition, rapid weight gain during infancy is consistently associated with increased subsequent risk for obesity [5]. Although the mechanisms behind the putative pathophysiological link between pre- and postnatal nutrition and obesity have not yet been established, glucocorticoids (GC) have been proposed as possible mediators of the permanent programming of obesity, insulin resistance, and other metabolic dysregulations [6].

GCs regulate, among other processes, adipose tissue differentiation, cellular lipid accumulation, and fat distribution. Alterations in central and peripheral GC activity have been described in both human obesity and animal models of obesity [7], [8]. At the tissue level, GC exposure is determined not only by circulating levels, but also by several enzymes that regulate local GC metabolism. In vivo, 11β-hydroxysteroid dehydrogenase type 1 (11β-HSD1) converts inactive GC to active GC (corticosterone or cortisol), which combine with glucocorticoid receptor (GR), and thus amplifies local GC action. 11β-HSD1 is mainly distributed in the liver, adipose tissue, and brain [9]. Adipose- or liver-specific 11β-HSD1 overexpression in transgenic mice exhibit elevated intra-adipose and portal corticosterone levels, abdominal obesity, dyslipidemia, insulin resistance, and hypertension [10], [11]. Previous studies showed higher 11β-HSD1 mRNA and/or activity in the adipose tissue of obese adults and children [12], [13], and 11β-HSD1 is positively associated with features of the metabolic syndrome in adults [14]. The principal enzymes responsible for GC catabolism in the liver are A-ring reductases (5α-reductase type 1 (5αR1) and 5β-reductase (5βR)). The activity and expression of these enzymes in the liver is higher in obese Zucker rats than in lean rats [8]; in humans, elevated hepatic 5αR1 activity is associated with insulin resistance [15] and both 5αR1 and 5βR activities are correlated with obesity [16]. 5αR1 is also expressed in adipose tissue, but is not dysregulated in rats or obese humans, at least not in subcutaneous fat [17]. The activities of both 11β-HSD1 and A-ring reductases can determine local tissue GC availability and also affect hypothalamic-pituitary-adrenal (HPA) axis activation; inhibition of 11β-HSD1 (e.g., decreased local GC generation) and activation of A-ring reductases (which enhances GC inactivation) may drive the HPA axis.

Studies have shown that local GC metabolism in adipose tissue and the liver is influenced by a number of factors, including age, nutrition, and hormones. Adipose tissue 11β-HSD1 expression [13] and enzyme activity (urinary terahydrocortisol [THF] and 5α-THF/tetrahydro-cortisone [THE] ratio) increases with age, particularly in boys [18]. GC and androgen hormones upregulate 11β-HSD1 [19] and growth hormone downregulates its expression [20]. Notably, GC and androgen appear to exert a depot-specific stimulation of the transformation of cortisone to cortisol within omental (i.e., visceral) adipose tissue [19]. Moreover, postnatal overfeeding of rats induces HPA axis hyperactivity and higher visceral 11β-HSD1 mRNA expression during adulthood [21]. Taken together, these observations strongly suggest the existence of a tight link between local GC production and hormone levels, age, and nutrition; this led us to hypothesize that the ontogeny of 11β-HSD1 in adipose tissue and liver and A-ring reductases in the liver is regulated by early postnatal overfeeding, and that puberty is a key phase of development in aberrant GC metabolism.

## Methods

### Animals and experimental design

All of the studies were approved by the University Committee on Use and Care of Animals, and were overseen by the Unit for Laboratory Animal Medicine of Nanjing Medical University (ID: 2008031801). Sprague-Dawley rats (Nanjing, Jiangsu, China) were maintained under controlled light (0600–1800 h) and temperature (22±2°C) conditions with free access to tap water and standard pellet diet.

Female rats were time-mated, and at postnatal day 3 (P3) male rat pup litters were randomly distributed among the mothers to adjust to litter sizes of three (small litter, SL) or ten (normal litter, NL) to induce early postnatal overfeeding or normal feeding [22], [23]. After weaning (P21), the pups of both litter groups were fed standard chow (15% calories as fat, 21% calories as protein and 64% calories as carbohydrate); all animals were housed three per cage post-weaning. Body weight, body length (nose to anus length) and food intake were monitored throughout life. As the puberty of rats is usually defined from postnatal days 21 to 60 [24], the animals were killed at postnatal weeks 3 (W3), 4, 5, 6, 8, and 16 between 0830 h and 1000 h after fasting overnight.

### Serum and tissue collection

Rats were anesthetized with chloral hydrate (300 mg/kg body weight, i.p.) at 0830 h after overnight fasting (12 h) and blood samples were obtained from the right ventricle. The blood was centrifuged (2,000× *g*, 4°C, 15 min) and the separated serum was stored at −70°C for subsequent determination of serum insulin, corticosterone, and leptin. Epididymal and retroperitoneal white adipose tissue and liver samples were removed, snap-frozen in liquid nitrogen, and kept at −80°C until gene expression and activity analysis. Adipose tissue was weighed and frozen, except for a portion of adipose tissue, which was fixed for determination of adipocyte cross-sectional areas [25].

### Histology

Adipose tissues were fixed in 10% formaldehyde in phosphate buffered saline (PBS), pH 7.4, for 24–48 h at room temperature. After fixation, tissues were dehydrated in graded ethanol, cleared in xylol, and embedded in paraffin at 60°C. The sections of 8 µm cut on the microtome (Leica, Wetzlar, Germany) were stained by hematoxylin and eosin (H/E), then analyzed with image software. At least 3 slices per adipose tissue sample, and 10 fields of vision each slice were accomplished to determine adipocyte cross-sectional areas.

### Biochemical analysis

Serum glucose was determined with a hexokinase assay (Thermo Electron, Melbourne, Australia), triglyceride and cholesterol were measured using an Olympus AU400 analyzer with enzymatic reagents (Olympus America, New York, USA). Insulin concentration was determined by a radioimmunoassay (BNIBT, Beijing, China) according to company instructions, with a sensitivity limit of 10 pmol/L and an intra-assay coefficient of variation of 10% for the assay. Serum corticosterone (Cayman, Michigan, USA) and leptin (Millipore, Billerica, USA) was measured by enzyme-linked immunosorbent assays, sensitivity limits were 40 pg/ml for corticosterone and 0.04 ng/ml for leptin, and the intra-assay coefficients of variation for corticosterone and leptin were 7–9% and 2–3%, respectively.

### Intraperitoneal glucose tolerance test measurement

The intraperitoneal glucose tolerance test (IPGTT) was performed as previously described [26]. Briefly, at W16 rats were fasted overnight, a fasting blood sample was taken from a tail vein, and then the rats were injected with 2.0 g D-glucose (50% stock solution in saline)/kg body weight intraperitoneally. Thereafter, blood samples were drawn at 30-, 60- and 120-min intervals after the glucose injection, and glucose levels were measured by a glucose meter (Accu-Chek; Roche, Mannheim, Germany).

### Quantitative real-time PCR

Total RNA was isolated from adipose tissue and liver with Trizol (Invitrogen, Carlsbad, CA, USA) according to the manufacturer's instructions and quantified spectrophotometrically at OD260. The integrity of total RNA was assessed using agarose gel electrophoresis, and cDNA was synthesized using M-MLV reverse transcriptase (Promega, Southampton, UK) with 1.0 µg of the RNA sample as described by the manufacturer. PCR amplification using glyceraldehyde-3-phosphate dehydrogenase (GAPDH) primers on a subset of the cDNA samples confirmed successful reverse transcription. Real-time PCR was performed using the SYBR GREEN ABI Prism 7500 sequence detector with cycling parameters of 50°C for 2 min, 95°C for 10 min, 40 cycles of 95°C for 15 sec, and 60°C for 1 min. The mRNA levels were normalized to the corresponding GAPDH mRNA levels. Data were analyzed with the 2^−ΔΔct^ method [27]. The sequences of the primers are in Table 1.

**Table 1:** Primer sequences used for mRNA quantification by real-time PCR.

	Forward primer 5′–3′	Reverse primer 5′–3′
11β-HSD1	GAA GAA GCA TGG AGG TCA AC	GCA ATC AGA GGT TGG GTC AT
5αR1	CTG TTT CCT GAC AGG CTT TGC	GCC TCC CCT GGG TAT CTT GT
5βR	GCC TTT AAG CCT GGA GAG GAA	ACG TGG CAC ACA GAT TTG ATT
GR	GGG TAC TCA AGC CCT GGA ATG	CCC GTA ATG ACA TCC TGA AGC T
C/EBPα	GGC GGG AAC GCA ACA A	TCC ACG TTG CGC TGT TTG
C/EBPβ	TCT ACT ACG AGC CCG ACT GC	AGG TAG GGG CTG AAG TCG AT
GAPDH	CAA GTT CAA CGG CAC AGT CAA	TGG TGA AGA CGC CAG TAG ACT C

11β-HSD1, 11β-hydroxysteroid dehydrogenase type 1; 5αR1, 5α-reductase type 1; 5βR, 5β-reductase; GR, glucocorticoid receptor; C/EBPα, CCAAT/enhancer-binding protein α; C/EBPβ, CCAAT/enhancer-binding protein β; GAPDH, glyceraldehyde-3-phosphate dehydrogenase.

### 11β-HSD1 enzyme activity

To estimate 11β-HSD1 protein, 11β-HSD1 activity was measured in adipose and hepatic tissue in the direction of the dehydrogenase reaction, because it is more stable in vitro than the reductase direction [28]. 11β-HSD1 enzyme activity measurements were performed essentially as previously described [29]. Briefly, tissue was homogenized in homogenization buffer (10% glycerol, 300 mM NaCl, 1 mM EDTA, and 50 mM Tris base pH 7.7) containing 1 mM dithiothreitol and then centrifuged at 4°C. Protein concentration was determined using a Pierce BCA protein assay kit with bovine serum albumin as the standard (Thermo Fisher Scientific, Rockford, IL, USA). Each sample was analyzed in duplicate, with the use of an internal control. Samples of adipose tissue (0.5 mg protein/ml) or liver (10 µg protein/ml) homogenate were incubated with 2 mM NADP and 100 nM 1,2,6,7-[^3^H]_4_-corticosterone (Amersham, Berkshire, UK). After incubation in a shaking water bath at 37°C for 2 h or 1 h for adipose tissue or liver, respectively, the reaction was interrupted; steroids were extracted with ethyl acetate and then dried, dissolved in ethanol, separated by thin-layer chromatography (mobile phase chloroform∶ethanol [92∶8]), and exposed to a phosphorimager tritium screen (GE Healthcare Europe, Freiburg, Germany). The TLC plates were then scanned and quantified using a Typhoon scanner (GE Healthcare Europe). 11β-HSD1 activity was expressed as percentage conversion of corticosterone into 11-dehydrocorticosterone.

### Western blotting

GR content was determined by Western blotting. Adipose tissues were homogenized in ice-cold Radio Immunoprecipitation Assay (RIPA) buffer to make whole cell lysates. Protein concentrations were determined using a Pierce BCA protein assay kit with bovine serum albumin as the standard (Thermo Fisher Scientific, Rockford, IL, USA). Protein of 40 µg was loaded in each well of 10% SDS–PAGE, separated, and transferred onto nitrocellulose (NC) filter membranes (0.45 µm; Millipore, Billerica, USA) at 4°C. The membranes were blocked in 5% non-fat milk for 1.5 h at room temperature and then blotted with a primary antibody to GR or actin (Santa Cruz, California, USA) at 1∶250 dilution overnight at 4°C. After being washed four times (10 min/wash) in PBST buffer at room temperature, the membrane was incubated with the second antibody for 1 h and immunoreactive bands were visualized using chemiluminescence. The intensity of target proteins (GR) and reference proteins (Actin) were quantified using Gel-Pro analyzer 4.0 software and the relative blackness of target proteins over reference protein was used to estimate the expression of GR.

### Statistical methods

Results are expressed as mean ± SEM. Significant differences among different ages in each group were analyzed by using one-way analysis of variance (ANOVA) followed by post-hoc Fisher's least significance difference (LSD) test. Differences between SL and NL groups at corresponding time points were analyzed by unpaired Student's *t*-test. Serum glucose during IPGTT was analyzed using repeated-measures ANOVA followed by Fisher's test. *P*<0.05 was considered to be significant. For all statistical evaluations, SPSS for Windows was used (version 13.0 from SPSS, Chicago, IL, USA).

## Results

### Effects of early postnatal overfeeding on body weight

**Figure 1:**
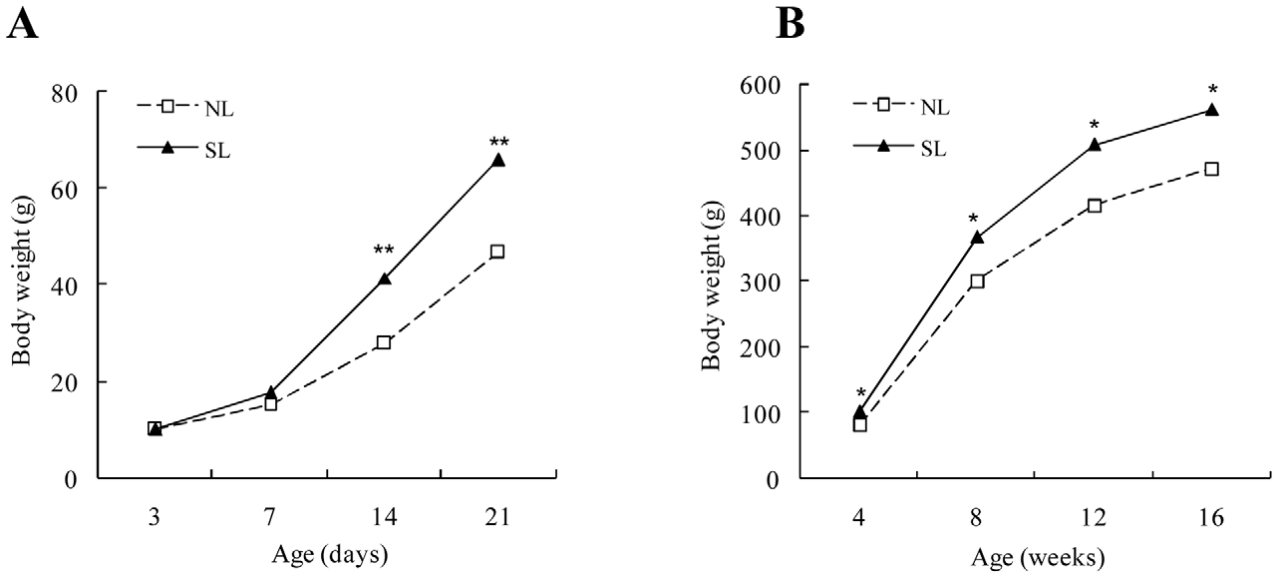
Body weight during the suckling (A) and post-suckling (B) periods in normal litters (NL) or small litters (SL) rats. Data are expressed as mean ± SEM. Differences between groups at the corresponding time points were analyzed by unpaired Student's *t*-test. *P<0.05, **P<0.01 for SL vs. NL rats at corresponding time points (*n* = 6–9).

**Figure 2:**
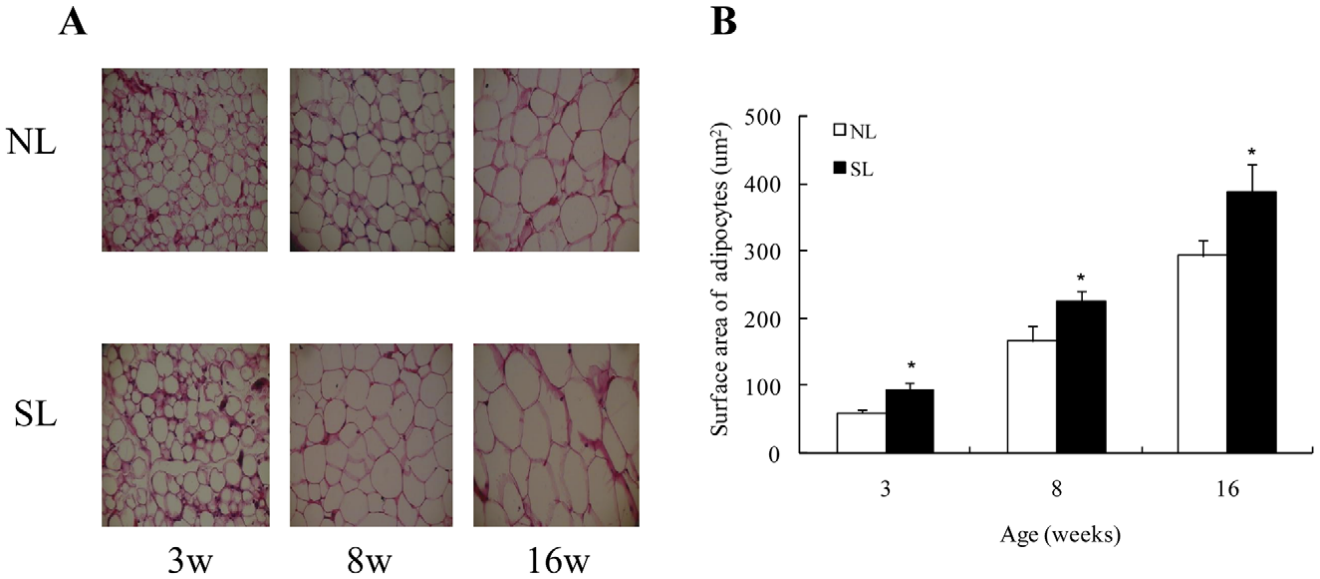
Hematoxylin-eosin–stained sections (400×) (A) and average adipocyte cross-sectional area (B) in retroperitoneal adipose tissue of NL and SL rats from postnatal week 3 (W3) to W16. Data are expressed as mean ± SEM. Differences between groups at the corresponding time points were analyzed using Student's unpaired *t*-test. *P<0.05 for SL vs. NL rats at the corresponding time point. (*n* = 6–9).

Body weight and fat pads increased with postnatal age in both groups. At postnatal day 14 (P14), body weight was higher in SL compared to NL rats (Fig. 1A); this persisted until W16 (Fig. 1B). The weight of retroperitoneal and epididymal fat pads was also higher in SL compared to NL rats from W3 to W16 (Table 2). When adjusted for body weight, retroperitoneal and epididymal fat weights in SL rats remained higher than in NL rats except for epididymal fat in W4 (Table 2). Throughout the study, the surface area of individual adipose cell increased with postnatal age in both groups (*P*<0.01), and SL rats had significantly higher average adipose cell cross-sectional areas than NL rats in both retroperitoneal and epididymal fat depots (Fig. 2A and 2B). There were no significant differences in body length between SL and NL rats from W3 to W16 (Table 3).

**Table 2:** Body fat weight (mg) at wk 3, 4, 5, 6, 8, and 16 in NL and SL rats.

	NL	SL
Epididymal fat (%BW)		
3 wk	136.73±18.27 (0.24±0.01)	198.35±19.05* (0.30±0.02*)
4 wk	240.87±17.89 (0.31±0.02)	300.00±12.82* (0.32±0.01)
5 wk	354.45±34.44 (0.26±0.03)	550.32±15.28** (0.33±0.01*)
6 wk	588.67±46.79 (0.29±0.02)	841.67±82.03* (0.36±0.01*)
8 wk	1638.96±168.76 (0.55±0.05)	2639.09±135.48** (0.72±0.04*)
16 wk	5943.76±471.32 (1.16±0.10)	7913.51±694.35* (1.41±0.07*)
Retroperitoneal fat (%BW)		
3 wk	131.33±11.79 (0.23±0.01)	228.33±37.37* (0.35±0.03**)
4 wk	248.32±22.84 (0.28±0.01)	305.63±11.54* (0.32±0.01*)
5 wk	318.29±45.87 (0.23±0.03)	620.37±50.70** (0.37±0.03**)
6 wk	554.33±78.06 (0.28±0.02)	838.33±26.00** (0.36±0.01**)
8 wk	1797.63±212.56 (0.59±0.05)	2751.31±134.63** (0.75±0.04*)
16 wk	6055.74±534.46 (1.18±0.11)	9457.53±614.52** (1.68±0.11**)

Data are expressed as mean ± SEM.

*P<0.05,

**P<0.01 for SL vs. NL. *n* = 6–9 in each NL and SL group.

%BW, % of body weight.

**Table 3:** Body length at wk 3, 4, 5, 6, 8, and 16 in NL and SL rats.

	NL	SL
Body length (cm)		
3 wk	13.88±0.24	14.2±0.31
4 wk	15.98±0.24	16.12±0.41
5 wk	18.01±0.35	18.33±0.29
6 wk	20.60±0.31	20.75±0.19
8 wk	24.17±0.27	23.58±0.32
16 wk	26.50±0.29	26.44±0.34

Data are expressed as mean ± SEM. *n* = 6–9 in each NL and SL group.

SL rats repeatedly ate more chow compared to NL rats, and there were significant differences in food intake between the two groups in W3 and W4 (Table 4).

**Table 4:** Food intake at wk 3, 4, 5, 6, 8, and 16 in NL and SL rats.

	NL	SL
Food intake (g/day)		
3 wk	7.70±1.13	10.60±1.24**
4 wk	11.65±3.33	14.32±2.18*
5 wk	17.45±2.32	17.56±1.23
6 wk	21.85±4.12	21.60±0.27
8 wk	25.17±1.01	25.60±1.87
16 wk	28.00±0.64	27.00±0.58

Data are expressed as mean ± SEM.

*P<0.05,

**P<0.01 for SL vs. NL. *n* = 6–9 in each NL and SL group.

### Effect of postnatal overfeeding on biochemical measurements

**Figure 3:**
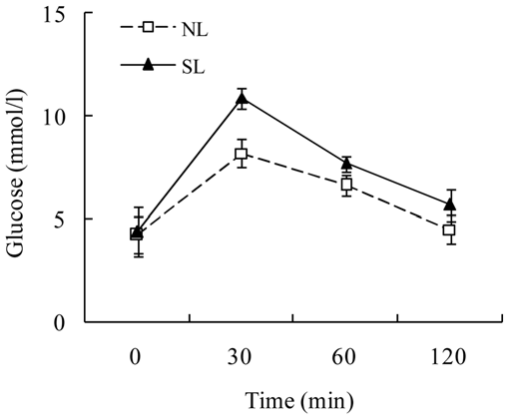
Intraperitoneal glucose tolerance test at W16 (2.0 g glucose/kg) in SL and NL rats. Data are expressed as mean ± SEM and were analyzed by repeated measures ANOVA followed by a post-hoc LSD test. *P<0.05, **P<0.01 for SL vs. NL rats at the corresponding time points (*n* = 6–9).

There were no differences in serum glucose, triglyceride and cholesterol levels from W3 to W16 (data not shown). Insulin levels at W16 were higher in SL rats compared to NL rats (108.65±4.88 vs. 87.06±6.48 pmol/L, *P*<0.05), and the insulin-to-glucose ratio was higher in SL rats than in NL rats (15.62±1.43 vs. 12.5±1.08, *P*<0.05). When given an intraperitoneal glucose load at W16, SL rats had higher circulating levels of glucose compared to NL rats at 30 and 60 min and a higher area under the curve, and this may indicate a lower insulin sensitivity or an impaired insulin secretion (Fig. 3).

### Circulating levels of corticosterone and leptin

**Figure 4:**
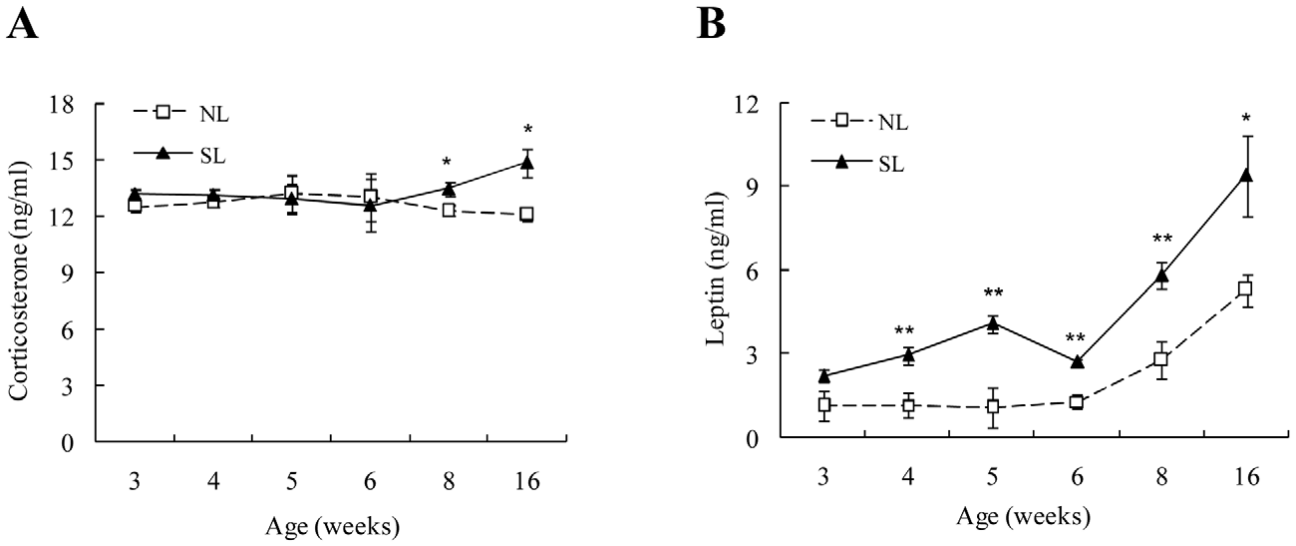
Serum corticosterone (A) and leptin levels (B) from W3 to W16 in SL and NL rats. Data are expressed as mean ± SEM. Differences between groups at corresponding time points were analyzed by unpaired Student's *t*-test. *P<0.05, **P<0.01 for SL vs. NL rats at the corresponding time points (*n* = 6).

The circulating corticosterone levels did not significantly change with postnatal age in the NL group, but increased in the SL group at W8 (although not at statistically significant levels) and W16 (*P*<0.05 vs. W3). W8 and W16 circulating corticosterone levels were higher in SL rats than in the NL rats at the corresponding time point (Fig. 4A). Serum leptin was increased during puberty in both groups (*P*<0.01), and SL rats exhibited significantly higher leptin levels from W4 to W16 (Fig. 4B) compared to NL rats.

### 11β-HSD1 mRNA expression and activity in adipose tissue of NL and SL rats

**Figure 5:**
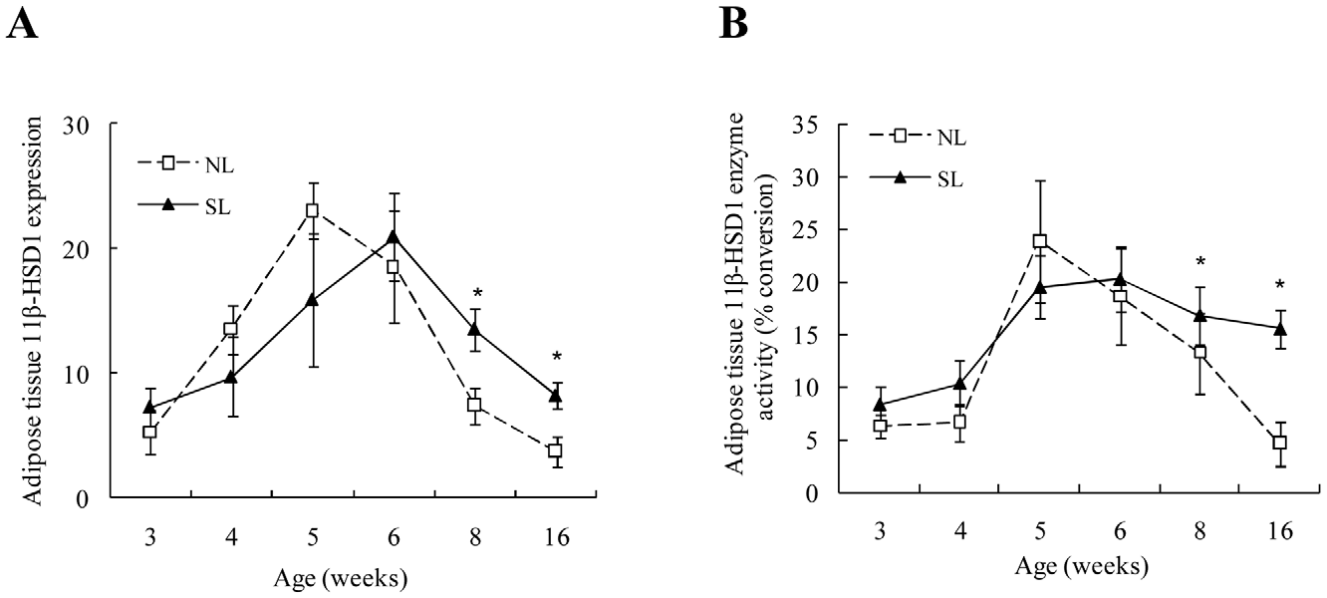
Retroperitoneal adipose tissue 11β-HSD1 mRNA expression (A) and enzyme activity (B) from W3 to W16 in SL and NL rats. Data are expressed as mean ± SEM. Differences between groups at corresponding time points were analyzed by unpaired Student's *t*-test. *P<0.05 for SL vs. NL rats at the corresponding time points (*n* = 6–9).

In retroperitoneal adipose tissue, 11β-HSD1 mRNA expression increased with postnatal age in both NL and SL rats; the NL group peaked at W5 (*P*<0.05 vs. W3), while the SL group peaked at W6 (*P*<0.05 vs. W3). From W8 onward, 11β-HSD1 mRNA expression (Fig. 5A) and enzyme activity (Fig. 5B) remained higher in SL rats than NL rats. There were no significant differences in 11β-HSD1 mRNA expression and activity in epididymal fat.

### Hepatic 11β-HSD1 and A-ring reductases in NL and SL rats

**Figure 6:**
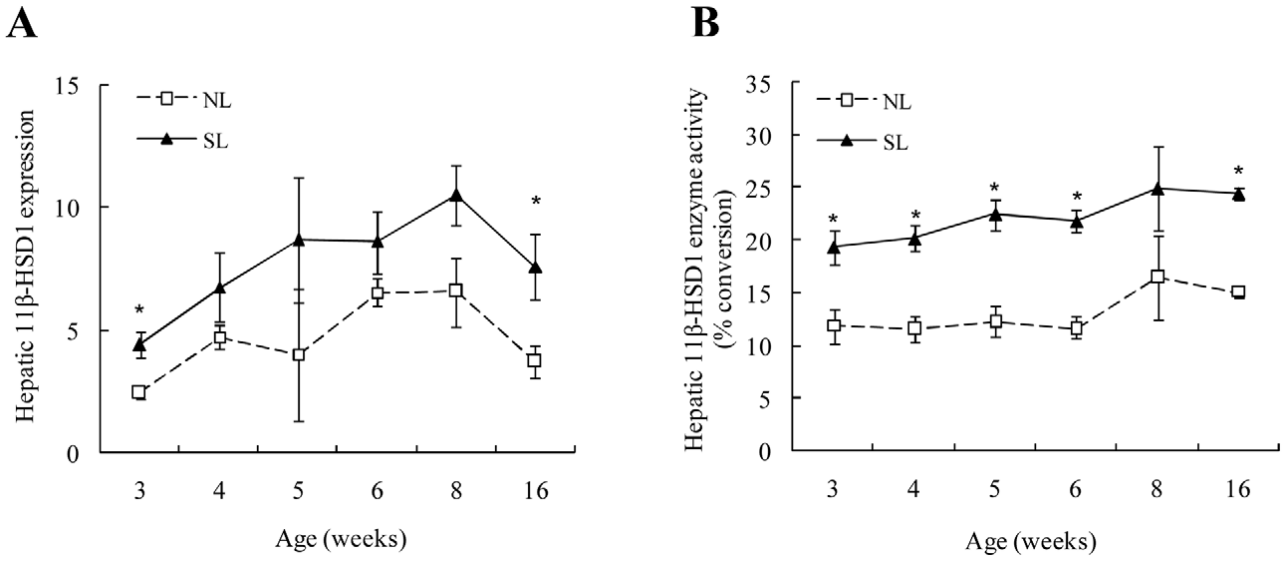
Hepatic 11β-HSD1 mRNA expression (A) and enzyme activity (B) from W3 to W16 in SL and NL rats. Data are expressed as mean ± SEM. Differences between groups at corresponding time points were analyzed by unpaired Student's *t*-test. *P<0.05 SL vs. NL groups at the corresponding time points (*n* = 6–9).

**Figure 7:**
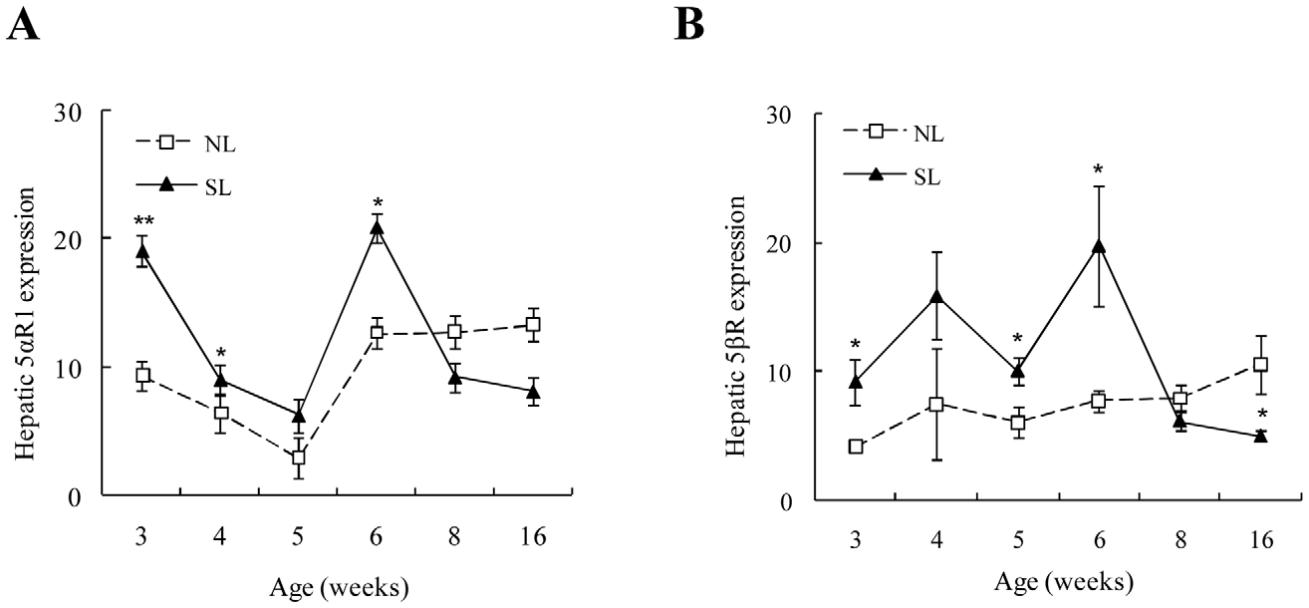
Hepatic 5αR1 (A) and 5βR (B) mRNA expression from W3 to W16 in SL and NL rats. Data are expressed as mean ± SEM. Differences between groups at corresponding time points were analyzed by unpaired Student's *t*-test. *P<0.05, **P<0.01 for SL vs. NL groups at the corresponding time points (*n* = 6–9).

11β-HSD1 mRNA expression and activity in the liver did not change with increasing postnatal age in either NL or SL rats. However, hepatic 11β-HSD1 mRNA expression was higher in SL rats compared to NL rats at W3 and W16 (Fig. 6A), and hepatic 11β-HSD1 activity was significantly elevated in SL rats from W3 to W16 compared to NL rats (except for W8) (Fig. 6B). Hepatic 5αR1 decreased with postnatal age and reached minimum levels at W5 in both the NL and SL groups (*P*<0.05 vs. W3) (Fig. 7A). 5βR mRNA expression did not significantly change with time in NL rats. Compared to the NL rats, hepatic 5αR1 (Fig. 7A) and 5βR mRNA expression (Fig. 7B) was higher in SL rats at W3, 4, and 6 and W3, 5, and 6, respectively, but decreased at W8, whereupon there were no apparent differences between the NL and SL groups. 5βR mRNA expression was actually lower in SL rats compared to NL rats in W16 (*P*<0.05).

### C/EBPα and C/EBPβ in adipose tissue of NL and SL rats

**Figure 8:**
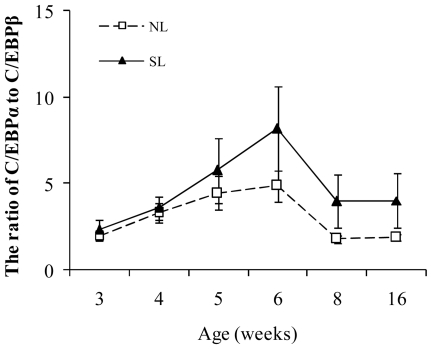
The ratio of C/EBPα to C/EBPβ mRNA expression in retroperitoneal adipose tissue from W3 to W16 in SL and NL rats. Data are expressed as mean ± SEM. Differences between groups at corresponding time points were analyzed by unpaired Student's *t*-test (*n* = 6–9).

CCAAT/enhancer-binding protein α (C/EBPα) has been shown to be a potent activator of the basal transcription of 11β-HSD1 whereas CCAAT/enhancer-binding protein β (C/EBPβ) is only a weak activator and competing with the strong activation of 11β-HSD1 promoter activity mediated by C/EBPα [30], [31], therefore, the transcriptional activity of 11β-HSD1 was assessed by the ratio of C/EBPα to C/EBPβ rather than measuring a single transcription factor alone. In retroperitoneal adipose tissue, the ratio of C/EBPα to C/EBPβ increased with postnatal age and peaked at W6 in SL (*P*<0.05) and NL (*P*<0.05) rats respectively. Although the C/EBPα to C/EBPβ ratio in SL rats was higher than in NL rats at every time-point, the difference did not reach significance (Fig. 8). There were no significant differences in the ratio of C/EBPα to C/EBPβ in epididymal adipose tissue with regard to either age or litter size (data not shown).

### GR mRNA and protein expression in adipose tissue and liver of NL and SL rats

There were no significant differences in GR mRNA and protein expression in liver, retroperitoneal and epididymal adipose tissue, with regard to either age or litter size (data not shown).

## Discussion

Manipulation of rat litter rearing during the postnatal period has been widely used to examine the effects of early overfeeding and has been shown to have long-term effects on body weight, appetite, and glucose tolerance [21], [22], [32]. Consistent with these reports, we confirmed that early postnatal overfeeding in rats can induce obesity in adults, as well as high circulating leptin and insulin resistance. Our most important finding was that peripheral 11β-HSD1 and 5αR1 and 5βR expression had different developmental patterns in SL rats compared to NL rats. 11β-HSD1 expression and activity in visceral adipose tissue was persistently higher from W8 afterward in SL rats compared to NL rats. Moreover, hepatic 11β-HSD1 expression and activity increased from W3 in SL rats, while A-ring reductase levels were higher in SL rats until W6, but the difference disappeared later in puberty (W8). At the same time (W8), the concentration of circulating corticosterone was increased in SL rats. These data suggest that early postnatal overfeeding induced by small litter rearing induces dysregulation of peripheral GC metabolism at puberty, which may contribute to obesity and metabolic alterations in adult rats.

In the present study, adipose tissue 11β-HSD1 expression appeared to be depot-specific in both SL and NL rats, since 11β-HSD1 expression was increased with age before puberty in retroperitoneal fat (visceral adipose tissue), but not in epididymal fat (which is not visceral), consistent with studies of this gene in sheep and children [13], [33]. Adipose tissue depot-specific dysregulation of 11β-HSD1 has been reported by others [19], [34] and may involve depot-specific promoter activation of C/EBPs [35]. In the present study, the ratio of C/EBPα to C/EBPβ increased with postnatal age in retroperitoneal adipose tissue, while no change was seen in epididymal adipose tissue. However, there were no difference in the ratio of C/EBPα to C/EBPβ between SL and NL rats, and other transcription factors may thus be involved in this process and this needs further research. The increase in visceral adipose 11β-HSD1 activity with age may contribute to local activation of GC, which in turn may promote preadipocyte differentiation and lipid accumulation [9], suggesting an important role for 11β-HSD1 in fat accumulation and distribution during development.

Interestingly, there were different developmental patterns in visceral adipose 11β-HSD1 expression and activity after puberty onset in NL compared to SL rats. 11β-HSD1 in NL rats began to decline earlier (W6) than in SL rats; thus, SL rats had higher visceral adipose 11β-HSD1 activity from W8 to adulthood. A large number of animal models have confirmed that high expression of 11β-HSD1 in visceral fat is closely related to obesity, insulin resistance, and lipid metabolism dysregulation. Thus, it is possible that overfeeding during lactation may underlie the increase in visceral fat GC activity at puberty and make SL rats more prone to fat accumulation and obesity, similar to what is observed in adipose tissue-selective overexpression of 11β-HSD1 in a transgenic mouse model [10]. However, the regulatory mechanisms of adipose 11β-HSD1 activity in SL rats during puberty is unclear, it is plausible that the factors derive from the adipose tissue itself, e.g. through different adipocytokines [36], [37], but this matter can only be concluded though future, more mechanistic, studies.

We found no differences in circulating corticosterone levels between the two groups in the earlier stages of puberty, and this correlates with a lack of change in GR expression in the liver. Importantly, we found that hepatic 11β-HSD1 mRNA was increased at W3 and W16 whereas activity was increased in SL rats from weaning to adulthood, as reported previously in db/db mice [38] and marmosets prenatally programmed with GC [39]. In the majority of obesity animal models, however, hepatic 11β-HSD1 expression and/or activity are unchanged [40] or downregulated [41]. This inconsistency may due to different time windows of exposure of overnutrition. Our animal model underwent general nutritional excess during lactation, while other obese animal models are usually manipulated in later puberty or later on [40], [42]. Perinatal nutrition may thus be pivotal in regulating hepatic 11β-HSD1 development. Mouse transgenic overexpression of 11β-HSD1 in the liver results in insulin resistance, dyslipidemia, hypertension, and fatty liver [11]. The increase in liver 11β-HSD1 may therefore be important for the development of insulin resistance in the present study. However, biochemical measures of serum triglycerides and cholesterol did not reveal any differences between the groups. The reason behind this inconsistency is currently unknown.

Circulating GC is inactivated by A-ring reductases in the liver. Studies of obese animals [8], [43] and humans revealed higher expression of hepatic 5α-reductase and normal circulating GC [15], while in hypertension patients, 5βR activity was decreased [44]. In the present study, SL rats showed higher hepatic A-ring reductase expression compared to NL rats in prepuberty. Higher A-ring reductase actvity may consequently increase GC clearance and lead to a compensatory activation of the HPA axis and reactivation of GC by 11β-HSD1 in the liver. However, the differences in A-ring reductase expression disappeared at the end of puberty and serum corticosterone was elevated. Since elevated GC actions influence pathways of hepatic glucose metabolism [45], this may aggravate metabolic disorders in SL rats. Taken together, the developmental ontogeny of GC metabolism in the liver could reflect the pathological process of metabolic disease in SL. Little is known about the regulatory mechanisms behind 5αR1 and 5βR expression during postnatal development. Androgens are potent regulators of 5αR1 [46], [47], and temporal 5αR1 expression during development may reflect the action of sex hormones, as W5 is near the onset of puberty and is accompanied by increasing androgen hormone levels [48]. Thus, investigating the developmental changes in liver 5αR1 in castrated rats is warranted in future research.

It is well known that variations in maternal care affect the development of individuals in neuroendocrine stress systems, e.g. the HPA-axis, in rats [49]. In our study, there may be subtle differences in mother-pup interactions such as maternal licking and grooming between SL and NL rats. Therefore, other factors beside nutritional factors may be involved in the changes in GC metabolism, and this needs further studies.

In conclusion, small litter rearing in rats can induce obesity and alter GC metabolism in adults. During puberty, increased 11β-HSD1 activity and decreased 5αR1 and 5βR expression in peripheral tissue can to contribute to dysregulated GC metabolism and hyperactivity of the HPA-axis, and may aggravate the pathological processes associated with nutritional programming in obese organisms. Furthermore, the developmental patterns of GC metabolism suggest that early interventions should be considered and brought into action before the onset of puberty.
